# Supplementary material for: Duchenne Muscular Dystrophy Patient iPSCs—Derived Skeletal Muscle Organoids Exhibit a Developmental Delay in Myogenic Progenitor Maturation
Source: Cells. 2025 Jul 7;14(13):1033. doi: 10.3390/cells14131033 (PMC12249143; doi:10.3390/cells14131033)
Supplement: Supplementary file 1 [file cells-14-01033-s001.zip › cells-3621956-supplementary.pdf]

cells

MDPI

Supplementary Materials: The following supporting information can be downloaded at: https://www.mdpi.com/article/10.3390/cells14131033/s1, Figure S1: Morphological characterization of SMO from hiPSCs. Figure S2: Quality control of organoid datasets. Figure S3: Expression of collagenase and proinflammatory markers in clusters of DMD1 and WT2 FAPs. Figure S4: Developmental maturation scores of myogenic progenitors in WT and DMD organoids. Figure S5: Immunohistochemistry characteriza-tion of DMD1 SMOs. Figure S6. Validation of the myogenic identity of SMOs. Table S1: Raw metrics of scRNA-seq datasets of WT and DMD organoids. Table S2: Detailed information of DMD hiPSCs. Table S3: Proportions of SMOs. Table S4: Differential expression of myogenic progenitors WT1 vs DMD1. Table S5: Maturation score MP comparison of WT and DMD. Table S6: Supplements for SMO media. Table S7: Abbreviations

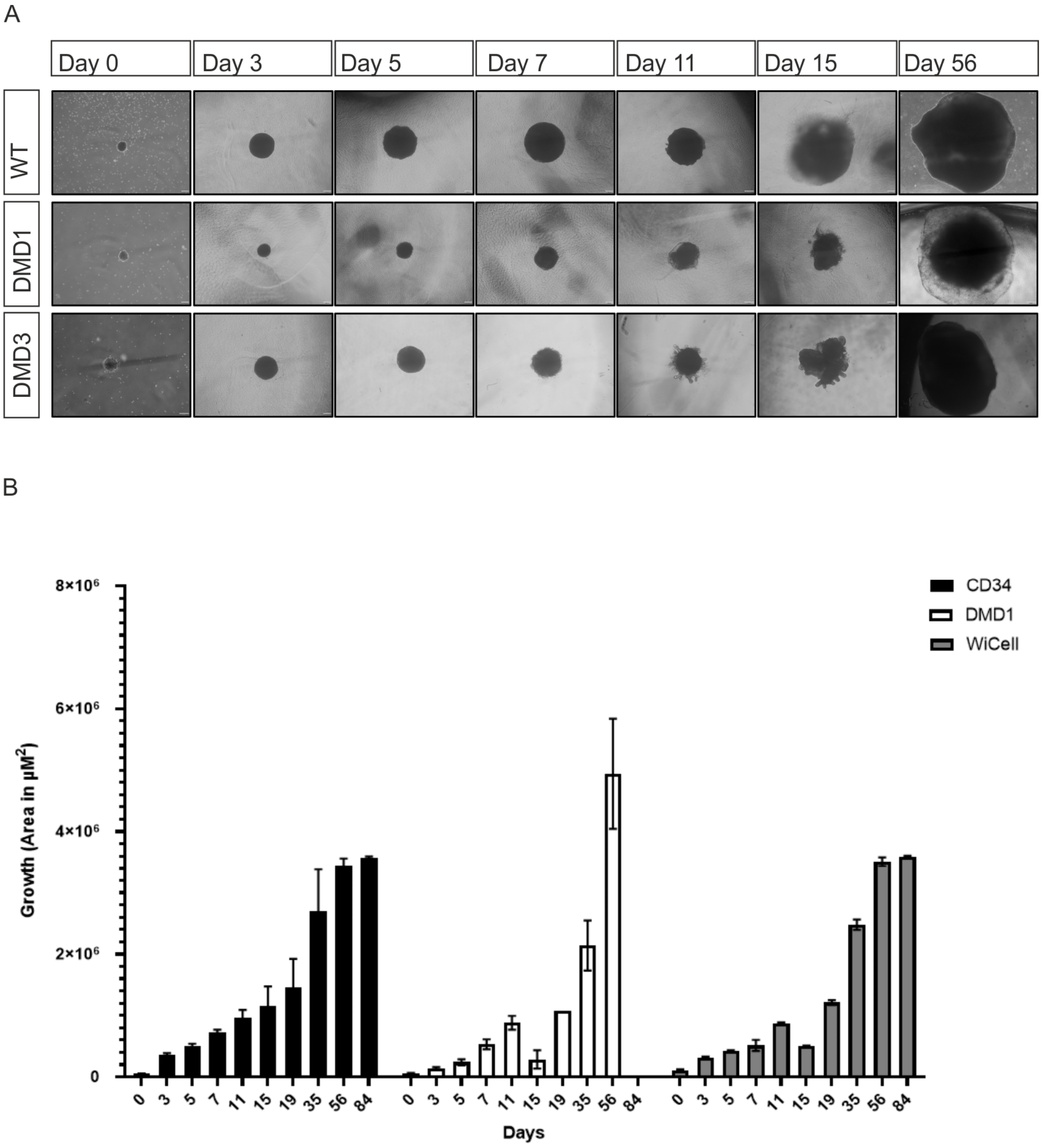

Figure S1. Morphological characterization of SMO from hiPSCs. (A) phase contrast microscopic images of organoid morphology at different time points of differentiation. Scale bar 50μm at day 0, 200μm from day 3-56. (B) Graphical depictions of SMO size. Mean area of organoids (in μm²) for selected days demonstrating average size (mean ± standard error of the mean (s.e.m.)) of SMO from WT ('CD34') and DMD1 and DMD3 (WiCell) lines.

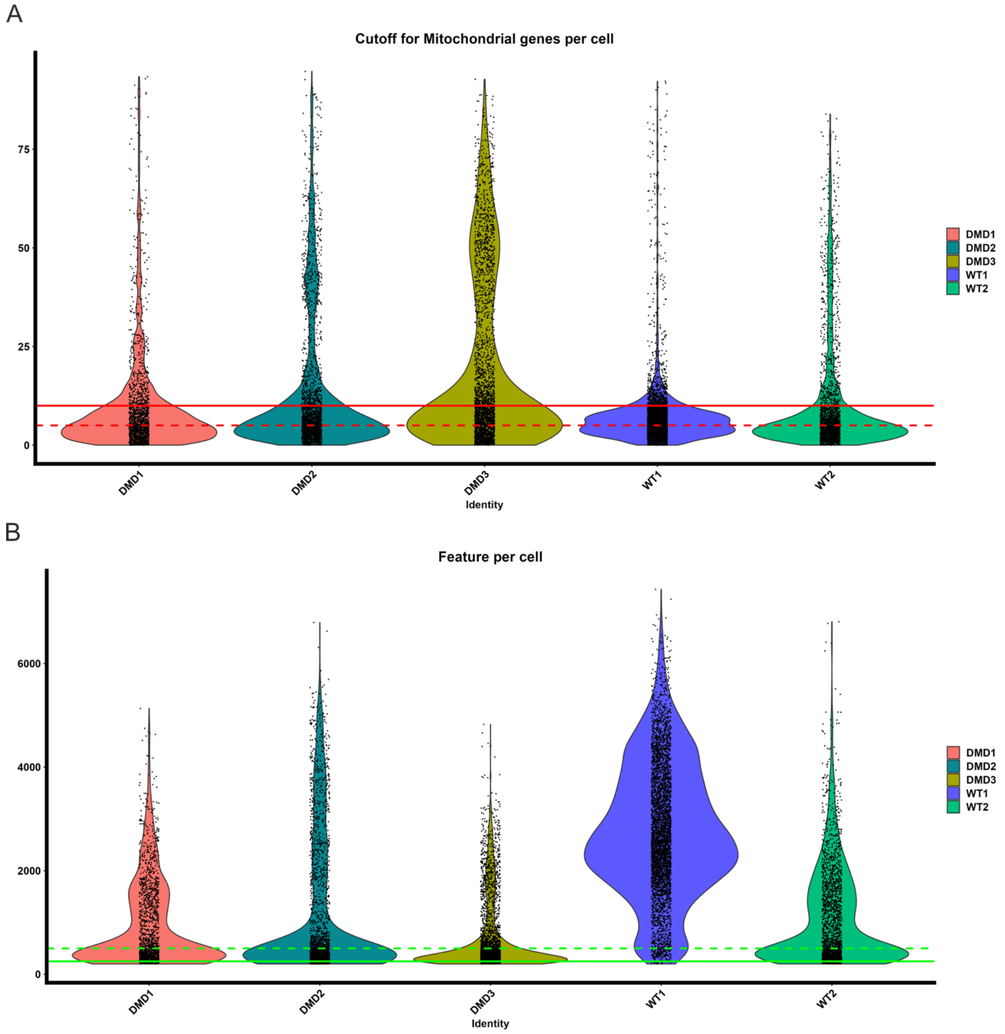

Figure S2. Quality control of organoid datasets. Violin plots from all organoid cell lines (WT1 - 2, DMD1 – 3) showing either mitochondrial or feature distribution. A) The percentage of mitochondrial genes in each cell of the dataset. The cutoff values are marked in red. the dashed line shows the value for dataset WT1. WT1 SMOs has a cutoff of 5% while WT2, DMD1-3 SMOs have a cutoff of 10%. B) The distribution of features of each cell is shown. The minimum cutoff of 500 features/ genes per cell for WT1 SMOs and 250 for WT2, DMD1-3 SMOs.

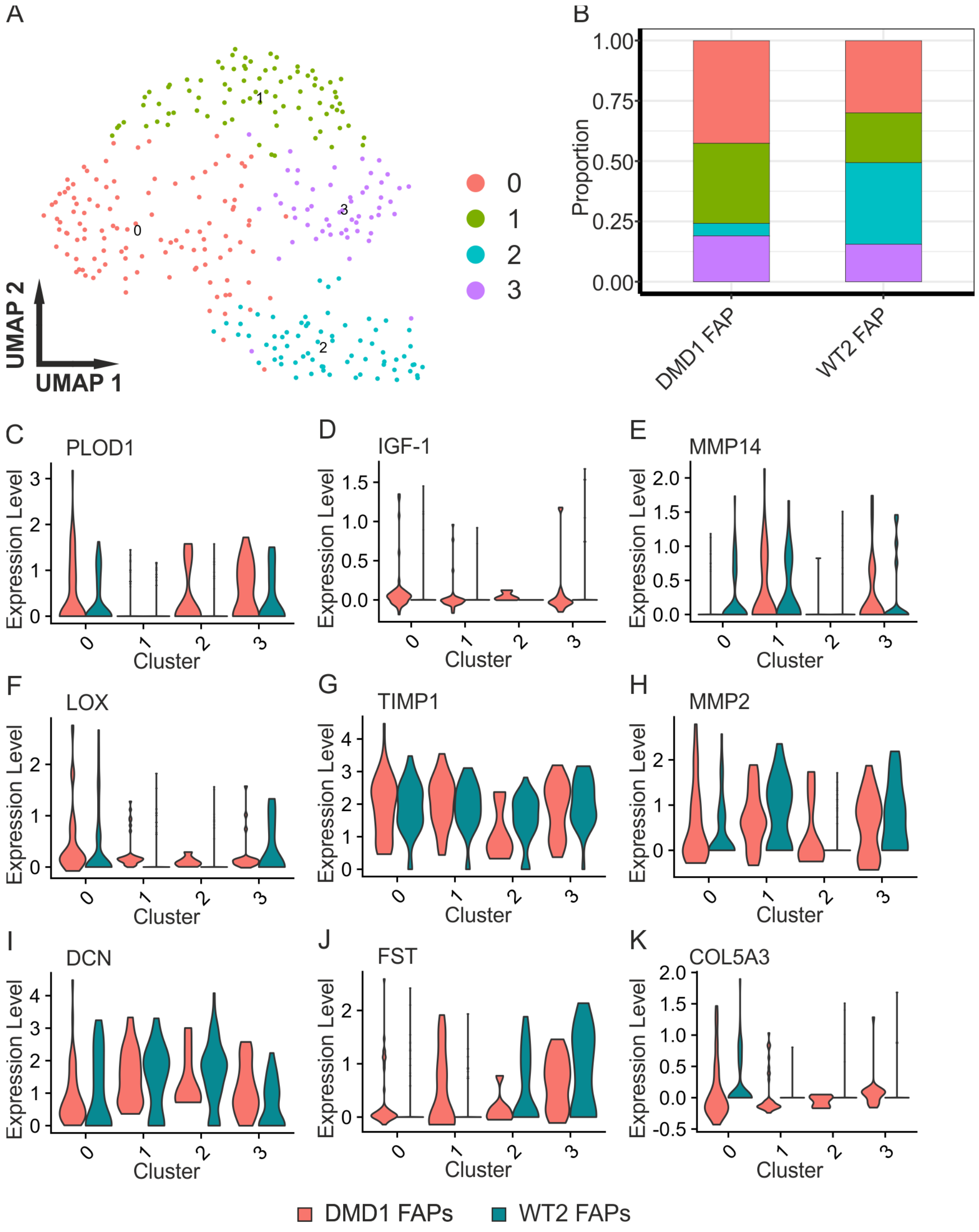

Figure S3. Violin plots expression pattern of collagenase and proinflammatory markers in clusters of DMD1 and WT2 fibro-adipogenic progenitors (FAPs). A) Cluster analysis of FAP subpopulations indicate 4 distinct clusters. B) The distribution within the FAP population indicates that cluster 0, 2 and 3 are more prominent in DMD as in WT. C)-K) Gene expression along the clusters is shown for PLOD1 (collagen synthesis marker), IGF-1 (general inflammatory marker) and MMP14, LOX, TIMP1, MMP2, DCN, FST and COL5A3 (proinflammatory markers). The expression is split into WT and DMD datasets.

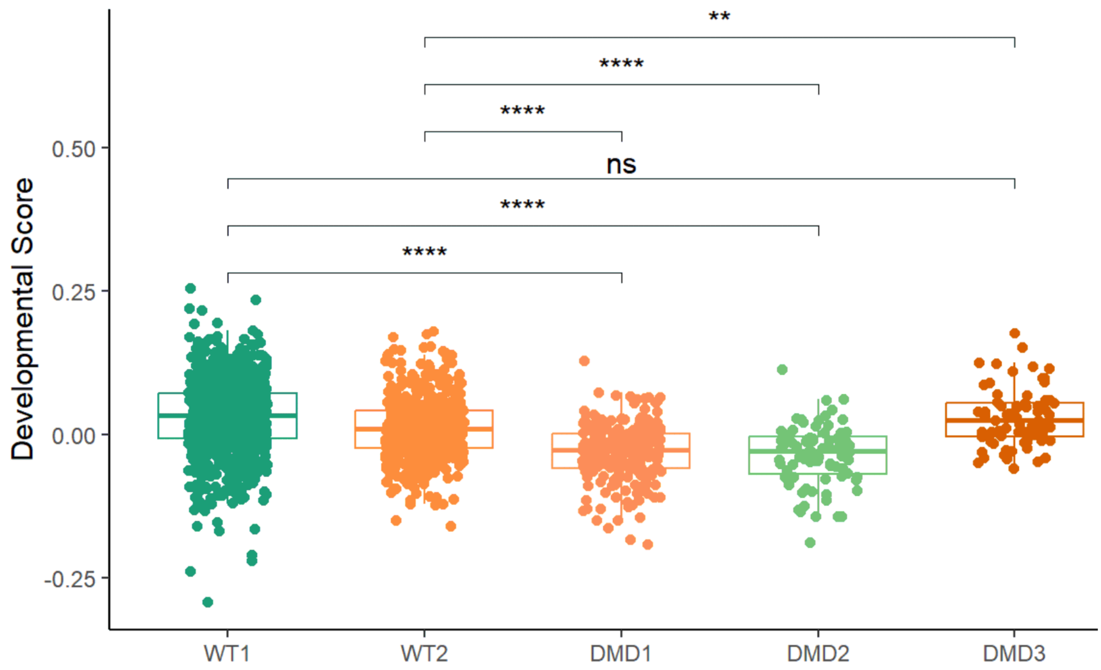

Figure S4. Developmental maturation scores of myogenic progenitors in WT and DMD organoids. Boxplots show developmental scores (y-axis) for myogenic progenitors derived from healthy (WT1, WT2) and DMD (DMD1, DMD2, DMD3) skeletal muscle organoids. Dots represent individual cells. Groups are color-coded: WT1 (teal), WT2 (green), DMD1 (orange), DMD2 (light orange), DMD3 (peach) (*: $p < 0.05$, **: $p < 0.01$, ***: $p < 0.001$ (see Supplementary Table S5 for exact p-values and effect sizes).

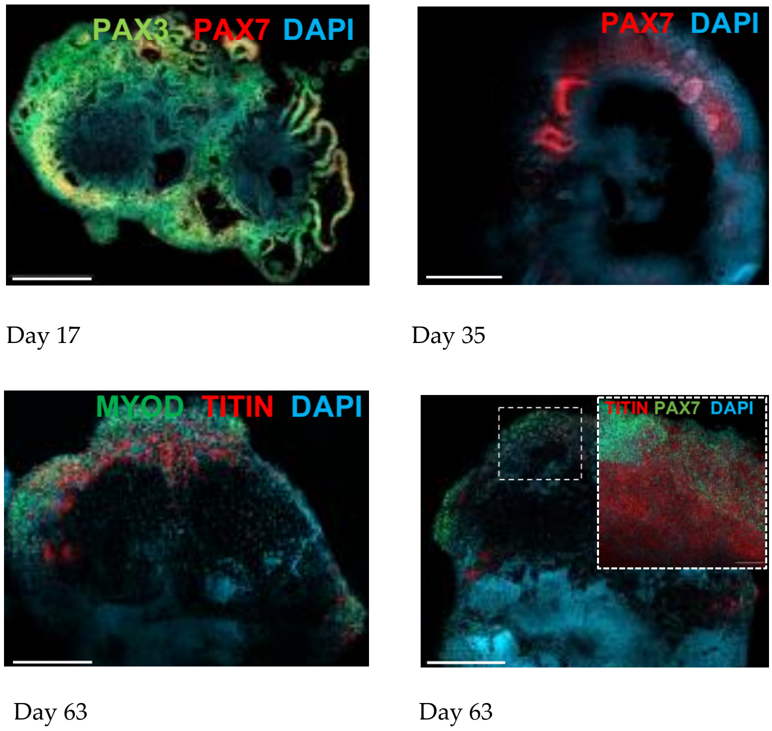

Figure S5. Immunohistochemistry characterization of DMD1 SMOs. Organoids from three distinct stages (Day 17, Day 35 and Day 63 after induction) according to Mavrommatis et al. (2023) [38] were fixed with paraformaldehyde, cryosectioned and incubated with primary antibodies anti-PAX3, anti-PAX7, anti-MYOD and anti-TITIN followed by secondary antibody stainings and imaging on a ZEISS LSM780 inverted confocal microscope (Material and Methods details in [38]). Scale bars: 100 µm (Day 17, 35), 200 µm (Day 63).

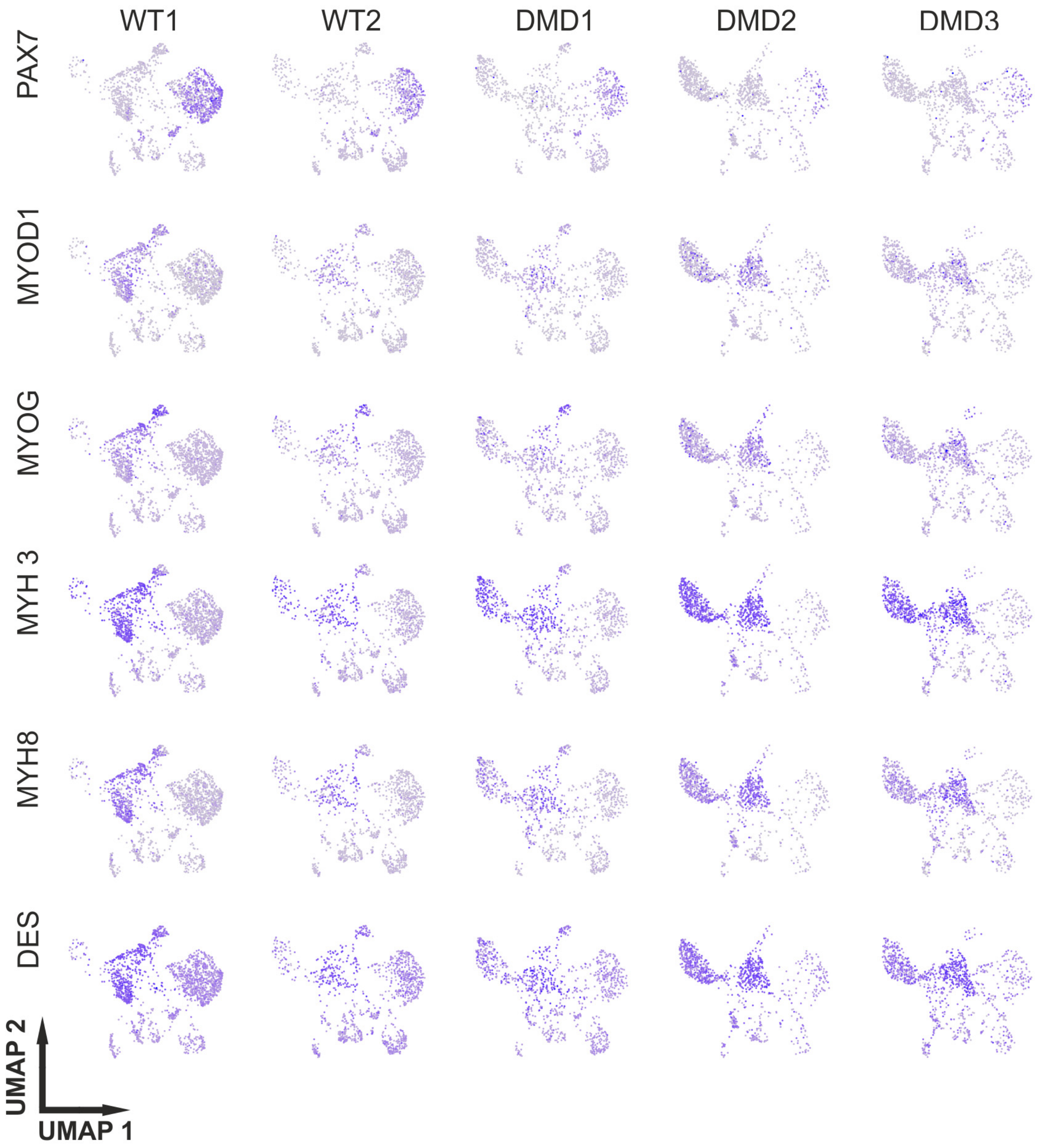

Figure S6. Validation of myogenic identity of SMOs. Feature plots showing expression of canonical muscle markers across integrated scRNA-seq data from healthy (WT1, WT2) and DMD (DMD1, DMD2, DMD3) SMOs corresponding to the clusters of Figure 1. Markers displayed in order: PAX7 (myogenic progenitor/ satellite cell identity), MYOD1 (myoblast determination), MYOG (terminal differentiation), MYH3 (embryonic myosin), MYH8 (neonatal myosin), DES (desmin, structural integrity).

Table S1. Raw Metrics of scRNA-seq Datasets WT and DMD organoids.

Sample	median genes per cell	estimated number of cells	Mean reads per cell	number of reads
WT1	2,585	4,666	37,018	172,726,184
WT2	618	2,486	90,493	224,965,946
DMD1	433	2,017	109,938	221,745,405
DMD2	419	2,771	83,833	232,303,207
DMD3	371	3,206	68,839	220,700,164

Table S2. Detailed information of DMD hiPSCs.

Line	Source	Age	Sex	Mutation	Phenotype	Reference
DMD1	Boston Children's Hospital	6	M	Ex45–52 del	DMD	[43]
DMD2	Boston Children's Hospital	6	M	Ex45–52 del	DMD	[43]
DMD3	UCSD/WiCell	23	M	Undisclosed	DMD + CMP	[44]
