# Supplementary material for: Duchenne Muscular Dystrophy Patient iPSCs—Derived Skeletal Muscle Organoids Exhibit a Developmental Delay in Myogenic Progenitor Maturation
Source: Cells. 2025 Jul 7;14(13):1033. doi: 10.3390/cells14131033 (PMC12249143; doi:10.3390/cells14131033)
Supplement: Supplementary file 1 [file cells-14-01033-s001.zip › Supplementary_Table_S7_Abbreviations.pdf]

Abbreviations and acronyms

General Abbreviations:

1. **DMD**: Duchenne Muscular Dystrophy
2. **BMD**: Becker Muscular Dystrophy
3. **SC(s)**: Satellite Cell(s)
4. **MP(s)**: Myogenic Progenitor(s)
5. **FAP(s)**: Fibro-Adipogenic Progenitor(s)
6. **ECM**: Extracellular Matrix
7. **SMO(s)**: Skeletal Muscle Organoid(s)
8. **iPSC(s)**: Induced Pluripotent Stem Cell(s)
9. **hiPSC(s)**: human Induced Pluripotent Stem Cell(s)
10. **scRNAseq**: Single-Cell RNA Sequencing
11. **DEG(s)**: Differentially Expressed Gene(s)
12. **HVG(s)**: Highly Variable Gene(s)
13. **MAD**: Median Absolute Deviation
14. **PC(s)**: Principal Component(s)
15. **UMAP**: Uniform Manifold Approximation and Projection

Gene/Marker Abbreviations:

1. **PAX7**: Paired Box 7
2. **MYF5**: Myogenic Factor 5
3. **MYOD**: Myogenic Differentiation 1
4. **MYOG**: Myogenin
5. **MYH3**: Myosin Heavy Chain 3 (embryonic isoform)
6. **MYH8**: Myosin Heavy Chain 8 (fetal isoform)
7. **NEB**: Nebulin
8. **SOX2**: SRY-Box Transcription Factor 2
9. **TOP2A**: Topoisomerase II Alpha
10. **PDGFRA**: Platelet-Derived Growth Factor Receptor Alpha
11. **CD44**: Cluster of Differentiation 44

12. **COL1A1/COL3A1**: Collagen Type I/III Alpha 1 Chain
13. **MMP2/MMP14**: Matrix Metalloproteinase 2/14
14. **TIMP1**: Tissue Inhibitor of Metalloproteinases 1
15. **LOX**: Lysyl Oxidase

Technical/Methodological Terms:

1. **CLR**: Centered Log Ratio (normalization method)
2. **FACS**: Fluorescence-Activated Cell Sorting

Other Terms:

1. **mTOR**: Mechanistic Target of Rapamycin
2. **IL-6**: Interleukin-6
3. **IGF**: Insulin-Like Growth Factor
4. **HGF**: Hepatocyte Growth Factor
5. **WNT**: Wingless/Integrated signaling pathway
6. **BMP**: Bone Morphogenetic Protein
7. **SHH**: Sonic Hedgehog
8. **FGF**: Fibroblast Growth Factor
